# Mental health in children and adolescents during the pandemic year 2020: Results from a department of child and adolescent psychiatry in Romania

**DOI:** 10.1192/j.eurpsy.2022.1105

**Published:** 2022-09-01

**Authors:** M. Opait, A. Floricel, L. Niculae, E. Andrei

**Affiliations:** Spitalul Clinic de Psihiatrie „Profesor Doctor Alexandru Obregia”, Child And Adolescent Psychiatry, Bucharest, Romania

**Keywords:** Covid-19, mental health, Children

## Abstract

**Introduction:**

Infectious diseases can disrupt the environment in which children live and have negative consequences for the well-being, development and mental health of this population.

**Objectives:**

Our aim is to compare the number and diagnoses of patients admitted during the first COVID-19 pandemic year to patients admitted during the same period in the previous year.

**Methods:**

Retrospective observational study of patients admitted between March 1, 2020 and February 29, 2021 and the analogous period of 2019-2020 in a child psychiatry unit. Microsoft Excel was used for descriptive statistics.

**Results:**

In our first pandemic year, there were 47.9% less admissions to our inpatient unit (n=717), comparing to the previous year (n=1376). Regarding the outpatient-type evaluations, there were 37.7% more admissions than the previous year (n=1813). Considering the fact that the number of inpatiens was limited in 2020 due to the restrictive measures imposed, most of our patients were consulted on an outpatient basis. During the 5 weeks nationwide lockdown, 72 children were consulted, 42.8% representing psychiatry emergencies. Regarding the diagnoses made in the whole pandemic year, disorders of psychological development were the most common, with almost half of the total of admissions (44.4%). The number of autistic outpatients in the pandemic year (n=1004) was almost double than the one before (n=572).

**Conclusions:**

Even though there was a reduction of admissions to the child psychiatry inpatient unit during the first pandemic year, we did not identify major differences regarding the discharge diagnoses comparing to the year before.

**Disclosure:**

No significant relationships.

